# Palliative Radiofrequency Ablation Accelerates the Residual Tumor Progression Through Increasing Tumor-Infiltrating MDSCs and Reducing T-Cell-Mediated Anti-Tumor Immune Responses in Animal Model

**DOI:** 10.3389/fonc.2020.01308

**Published:** 2020-09-04

**Authors:** Hao Wu, Su-shu Li, Meijun Zhou, An-Na Jiang, Yanni He, Song Wang, Wei Yang, Hongmei Liu

**Affiliations:** ^1^Department of Ultrasound, Institute of Ultrasound in Musculoskeletal Sports Medicine, Guangdong Second Provincial General Hospital, Southern Medical University, Guangzhou, China; ^2^Key Laboratory of Carcinogenesis and Translational Research (Ministry of Education/Beijing), Department of Ultrasound, Peking University Cancer Hospital & Institute, Beijing, China

**Keywords:** palliative radiofrequency ablation, myeloid-derived suppressor cells, tumor recurrence, immune response, residual tumor

## Abstract

Previous studies showed that radiofrequency ablation (RFA) has a favorable treatment efficacy for hepatocellular carcinoma (HCC) or colorectal liver metastases (CRLMs). Palliative RFA (pRFA) resulting from larger HCC or multiple CRLMs further accelerated the progression of potential residual tumor, yet its mechanism was still unknown. This study investigated the influence of myeloid-derived suppressor cells (MDSCs) on T-cell immune responses and tumor recurrence after pRFA. CT26 tumor models were used. The percentage of MDSCs in peripheral blood was analyzed by flow cytometry after pRFA. The level of Th1 and Th2 cytokines were measured by ELISA through different treatments (*n* = 4/group). The tumor-infiltrating MDSCs, dendritic cells, and intracellular cytokines level were analyzed by IHC staining after different treatments. The functional CD8^+^ T cells were confirmed by the co-localization immunofluorescence staining. The long-term outcomes were also evaluated through CT26 and 4T1 tumor models. The results showed that tumor models treated with pRFA displayed significant increases in the percentage of MDSCs of peripheral blood and tumor infiltration. The expression level of TGF-β and IL-6 after pRFA was higher than that before pRFA by ELISA and IHC staining. After depleting MDSCs by combining with Abs, the pRFA + Abs group achieved a higher level of Th1 cytokines and greatly enhanced the percentage of tumor-infiltrating functional CD8^+^ T cells when compared with pRFA alone. The depletion of MDSCs through combination with Abs also resulted in tumor regression. In conclusion, pRFA accelerates the residual tumor progression through increasing tumor-infiltrating MDSCs and reducing T-cell-mediated anti-tumor immune responses, which could provide a potential approach for delaying tumor recurrence caused by pRFA.

## Introduction

Radiofrequency ablation (RFA) has attracted increasing attention as a minimally invasive treatment for many tumors, especially hepatocellular carcinoma (HCC) and colorectal liver metastases (CRLM) (1, 2). Radiofrequency ablation has no inferior therapeutic effects when compared with liver resection for small HCC (≤5 cm) or solitary CRLM (3, 4). Previous studies in animal models have demonstrated that in addition to inducing local coagulation necrosis, RFA could also lead to the release of abundant amounts of cell debris, which can serve as the source of tumor antigens to induce anti-tumor immunity mediated by tumor-specific T cells (5–7). However, the antitumor immune response by RFA was insufficient to inhibit tumor recurrence and progression, even palliative RFA (pRFA) resulting from larger HCC or multiple CRLM further accelerated the progression of a potential residual tumor, but the mechanism causing this was unknown.

Recent studies have displayed that an ability to evade immune destruction plays an important role in tumor recurrence and progression (8–10). These studies also demonstrated that myeloid-derived suppressor cells (MDSCs), as immature immune suppressor cells mainly marked by Gr1^+^CD11b^+^ in mice, can be recruited and activated in the inflammatory condition and tumor microenvironment (TME) (11). In the TME MDSCs can directly or indirectly impair T cell-mediated immune responses, thus inhibiting the release of several anti-tumor cytokines, such as IFN-γ and TNF-α, and weakening immune destruction for tumors (12). Evidence from clinical and pre-clinical studies also confirmed that the proportion of MDSCs in peripheral blood or tumor tissues was inversely associated with survival, and that inhibiting the expansion and activity of MDSCs can significantly improve anti-tumor therapeutic effects (13–15). However, it is still unclear whether MDSCs are involved in regulating the anti-tumor Tcell immune responses induced by pRFA, leading to the residual tumor recurrence. Therefore, this study aimed to examine the influence of MDSCs on T cell-driven response and tumor recurrence after pRFA.

In this study, we firstly selected the murine CT26 and 4T1 cancer model commonly used in the immune experiment. We analyzed the influence of pRFA on the recruitment and intratumoral infiltration of MDSCs by flow cytometry and immunohistochemical staining. Meanwhile, we evaluated the effect of MDSCs on the T cell-driven response induced by pRFA through combining MDSCs depletion. The successful implementation of the findings of this study will provide an effective potential approach for delaying tumor recurrence after pRFA.

## Materials and Methods

The experimental animal protocol was first approved by the Institutional Animal Care and Use Committee of Peking University Cancer Hospital. In this study, the murine CT26 colon cancer model was used in the first two parts below, and the 4T1 and CT26 tumor models were used in the long-term follow-up. This study was employed in three phases to evaluate and analyze the influence of pRFA on the recruitment and intratumoral infiltration of MDSCs and the effect of MDSCs on tumor recurrence after pRFA, the details of RFA and the long-term outcomes from 4T1 tumor models are included in this article’s Supplementary Material.

### The Influence of pRFA on T Cells and MDSCs

Flow cytometry was performed to analyze the influence of RFA on the CD4, CD8, MDSCs via mice bearing CT26 colon carcinoma (*n* = 4). At –1, 3, and 8 days after RFA, 300 μL of blood for each one was harvested by canthus ophthalmic vein and transferred into heparinized tubes. Then the whole blood cells were stained by with FITC-conjugated CD45, PE-conjugated CD8a, APC-conjugated CD4, FITC-conjugated CD11b, PE-conjugated Ly6G/Ly6C (GR1). After 15 min, red blood cells lysate was added and then suspended in flow cytometry buffer. Finally, analysis was carried out by flow cytometry using a FACS flow cytometer (Becton Dickinson).

In order to analyze the percentage of tumor-infiltrating MDSCs, we performed immunohistochemical analysis. Firstly, tumor tissues were fixed in 10% formalin and embedded in paraffin in accordance with standard procedures. Sections were stained with anti-Ly-6G/Ly-6C antibodies (BioLegend, San Diego, CA, United States). The antibody was replaced with non-immune serum to serve as a negative control. After anti-Ly-6G/Ly-6C antibodies staining, observations were performed with a microscope for immunohistochemical staining analysis, and representative digital images were obtained.

### The Effect of MDSCs on Anti-Tumor Immune Response After pRFA Plus Abs

According to the manufacturer’s protocols, specific ELISA kits for the level of Th1 cytokines (IFN-γ and TNF-α) and Th2 cytokines (TGF-β and IL-1β) were used to measure cytokine levels in serum and conditioned media for different treatment groups (*n* = 4/group), including antibody alone (Abs), RFA alone, the combination of RFA with Abs (RFA + Abs).

To identify tumor-infiltrating dendritic cells (DC), immunofluorescence staining was performed by using a polyclonal American hamster PE-labeled CD11c antibody. The antibody was replaced with non-immune serum to serve as a negative control. After CD11c antibody staining, the slides were counterstained and mounted with a medium containing 4, 6-diamidine-2-phenylindole (DAPI). To further determine the function of CD8^+^ T cells, co-localization immunofluorescence staining was also carried out. After PE-labeled CD8 antibody staining, the slides were counterstained with FITC-labeled IFN-γ antibody. Representative digital images were obtained and analyzed by fluorescence microscope. Images were independently analyzed by two blinded evaluators. Each specimen was divided into 5 fields.

### Long-Term Outcomes

Thirty-two mice bearing CT26 tumors were used in this phase. Tumors measuring 10–12 mm in size were randomized into 4 experimental groups for tumor growth (*n* = 8/group). These groups included: (a) control (PBS only), (b)Abs, (c) RFA, and (d) RFA + Abs. The mice receiving PBS without RFA or Abs were used as the control. The tumor volume of each mouse was recorded every 3 days for three weeks. After finishing this experiment, tumors were harvested from mice, and then the weight of each tumor was measured.

### GR1 Depletion

Systemic GR1 depletion was achieved by intraperitoneal injection of 300 mg of purified monoclonal anti-Gr1 antibody RB6-8C5 (Abs, BioXCell, West Lebanon, NH, United States) at days – 1, 0, 3, 5, and 7 after RFA, and the volume of tumor was followed.

### Statistical Analysis

All continuous data were provided as means of standard deviations. The Student’s *t*-test and ANOVA were used to evaluate the measurement of data. The ANOVA for repeated measurement data was used to compare tumor size and tumor weight at different time courses between different groups. In all cases, *P* < 0.05 was considered statistically significant. SPSS 21.0 statistical analysis software (SPSS, Chicago, IL, United States) was used.

## Results

### The Influence of RFA on T Cells and MDSCs

**FIGURE 1:**
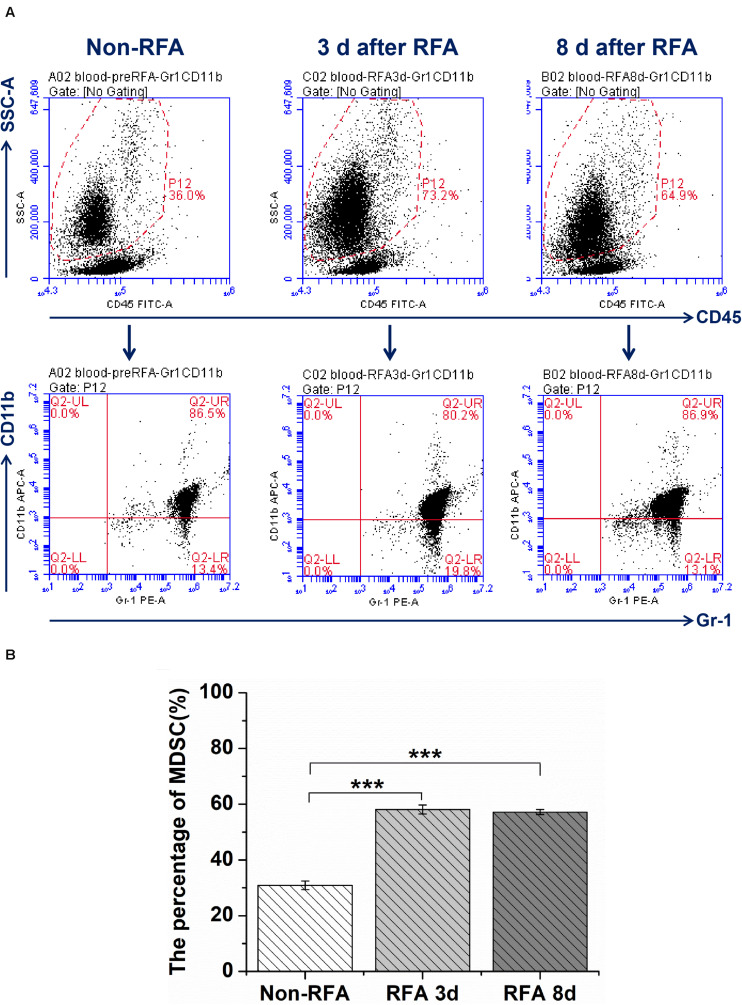
The flow cytometry analysis for MDSCs was performed through whole blood cells from mice bearing tumor treated with different treatment groups at different time points (Non-RFA, 3 days after RFA, 8 days after RFA). **(A)** the representative images from one experiment show a shift in the MDSCs population by flow cytometry analysis. **(B)** The percentage of MDSCs from different treatment groups at different time points, ***P < 0.001, **P < 0.01.

The results from flow cytometry analysis showed that the percentage of MDSCs in peripheral blood at 3 days after RFA was 1.9-fold higher than that before RFA (*P* < 0.001), and the percentage of MDSCs in peripheral blood had no significant change at 3 days and 8 days after RFA (*P* > 0.05) (Figures 1A,B). For the CD4/CD8 ratios, the ratio of CD4/CD8 at 3 days after RFA was lower than that at 1 day before RFA (6.15 vs 4.56, *P* < 0.001), and the ratio of CD4/CD8 at 8 days after RFA continued to decrease compared to that at 3 days after RFA (4.08 vs 4.56, *P* < 0.01) (Supplementary Figures S1A,B).

**FIGURE 2:**
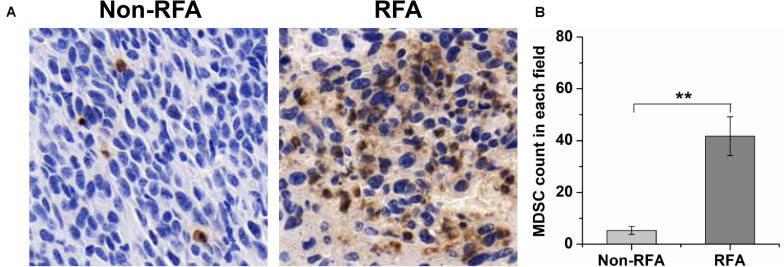
At 8 days after RFA, tumors were taken out and sectioned, and the Gr-1 staining was performed to analyze MDSC infiltration. **(A)** the representative plots about tumor-infiltrating MDSCs before or after RFA. **(B)** The quantitative analysis showed that the percentage of Gr-1 positive cells at 8 days after RFA was 10.4-fold higher than that before RFA (***P < 0.01).

To further investigate the influence of RFA on intratumoral MDSCs, immunohistochemical staining was used to analyze the tumor-infiltrating MDSCs by tumor sections from mice treated with different treatments. From the images of immunohistochemical staining, we found that RFA significantly increased the percentage of tumor-infiltrating Gr-1 positive cells (Figure 2A). The quantitative analysis showed that the percentage of Gr-1 positive cells at 8 days after RFA was 10.4-fold higher than that before RFA (*P* < 0.001) (Figure 2B).

### The Effect of Anti-Tumor Immune Response After pRFA Plus Abs

**FIGURE 3:**
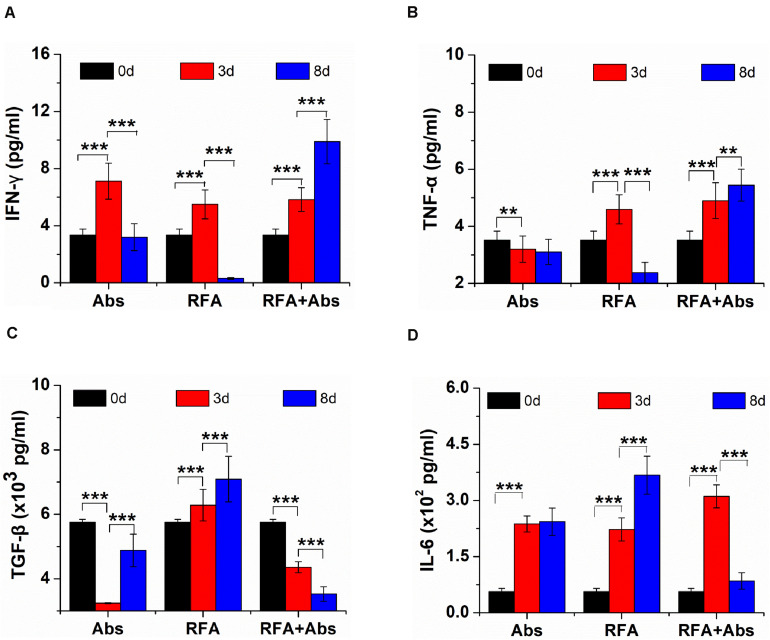
The ELISA assays were used to evaluate systemic immune responses by measuring the level of cytokine *in vivo* from different treatments (Abs, RFA, and Abs + RFA) at different time points (before RFA, 3 days after RFA, 8 days after RFA). **(A)** The ELISA assays of IFN-γ from different groups at different time points. **(B)** The ELISA assays of TNF-α from different groups at different time points. **(C)** The ELISA assays of TGF-β from different groups at different times. **(D)** The ELISA assays of IL-6 from different groups at different time points. ***P < 0.001, **P < 0.01.

The ELISA assays were used to evaluate systemic immune responses by measuring the level of cytokine *in vivo* in different treatments. The data showed that the level of IFN-γ increased in the first 3 days after RFA but decreased from 3 to 8 days after RFA alone. Meanwhile, the level of IFN-γ was on the rise within 8 days after RFA + Abs, and there were significant differences in the level of IFN-γ before RFA when comparing with that at 3 days and 8 days after RFA + Abs (*P* < 0.001) (Figure 3A). Moreover, the change in TNF-α (Figure 3B) was similar to that in IFN-γ for RFA alone group and RFA + Abs group. For TGF-β, the level of TGF-β was on the rise within 8 days after RFA alone, the level of TGF-β at 8 days after RFA alone was 1.23-fold higher than that before RFA (*P* < 0.001). However, in the RFA + Abs group, the level of TGF-β was declining within 8 days, and the level of TGF-β at 8 days after RFA + Abs was 1.63-fold lower than that before RFA alone (*P* < 0.001) (Figure 3C). For IL-6, the level of IL-6 was increasing within 8 days after RFA alone, and the level of IL-6 at 8 days after RFA alone was 6.55-fold higher than that before RFA (*P* < 0.001). When combining with Abs, the level of IL-6 at 8 days after RFA + Abs had no significant difference when compared with that before treatment (*P* > 0.01), but the level of IL-6 at 3 days after RFA + Abs had a certain increase when compared with that before treatment (*P* < 0.001) (Figure 3D).

Then the intratumoral expression level of TGF-β (Supplementary Figures S2A,B) and IL-6 (Supplementary Figures S3A,B) was analyzed by immunohistochemical staining to evaluate systemic immune responses *in vivo* through different treatment. The results from immunohistochemical staining showed that the expression level of TGF-β and IL-6 after RFA alone had a generally higher result than that in other groups, and the other three groups showed similar expression levels. The quantitative analysis also confirmed that there was a significant difference between the RFA alone group and the other three groups (*P* < 0.001).

**FIGURE 4:**
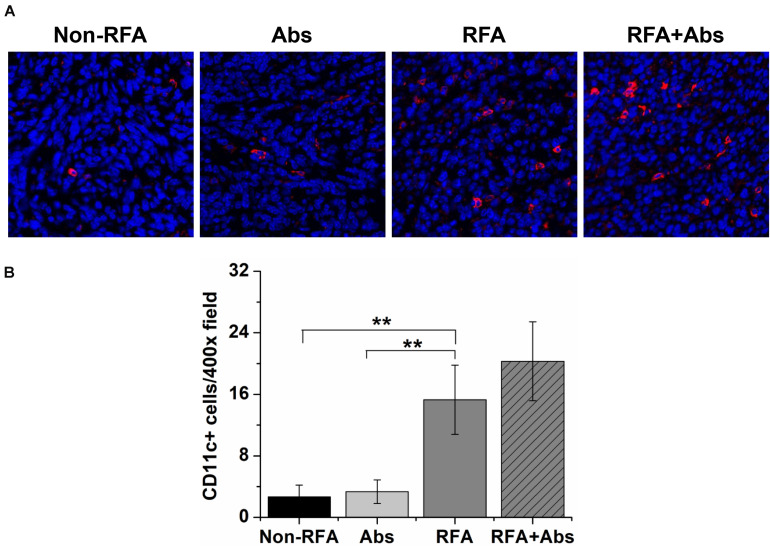
Immunofluorescence staining was performed by using a polyclonal American hamster PE-labeled CD11c antibody to identify tumor-infiltrating dendritic cells (DCs) after different treatment groups (Non-RFA, Abs, RFA, and RFA + Abs). **(A)** The representative images of immunofluorescence staining for DCs from different treatment groups, the red fluorescence indicates CD11c + cells, and the blue fluorescence indicates a cell nucleus; **(B)** the quantitative analysis of DCs from different treatment groups, the results displayed that both RFA alone group and RFA + Abs group enhanced the percentage of tumor-infiltrating DCs when compared with Abs alone group and control group (**P < 0.01).

Meanwhile, the results also displayed that both the RFA alone group and the RFA + Abs group had an enhanced percentage of tumor-infiltrating DCs. Further analysis showed that there was no significant difference between RFA + Abs and RFA alone (*P* > 0.05). Then, the results found that the injection of antibody alone did not increase the percentage of tumor-infiltrating DCs, and there was no significant difference between the Abs alone group and the control group (*P* > 0.05) (Figures 4A,B).

**FIGURE 5:**
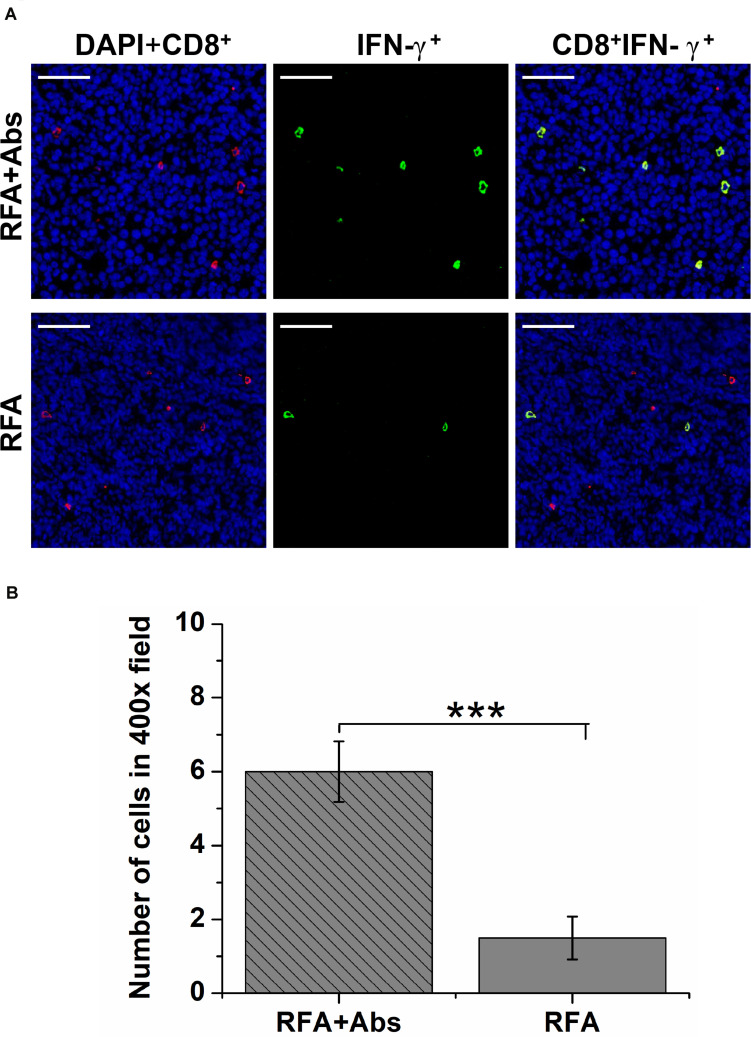
The double immunofluorescence staining was performed to analyze the percentage of IFN-γ^+^ CD8^+^ T cells to determine the functional tumor-infiltrating lymphocytes (TILs). **(A)** The representative images of double immunofluorescence staining for IFN-γ + CD8 + T cells from different treatment groups (RFA and RFA + Abs), the red fluorescence indicates CD8^+^ cells, and the green fluorescence indicates IFN-γ^+^ TILs; **(B)** the quantitative analysis of IFN-γ^+^ CD8^+^ T cells from different treatment groups, the results indicated that the percentage of IFN-γ + CD8 + T cells was significantly increased in the RFA + Abs group when compared with that in the RFA group (***P < 0.001).

To determine the tumor-infiltrating functional lymphocytes (TILs), double immunofluorescence staining was performed to analyze the percentage of IFN-γ^+^ CD8^+^ T cells. The results indicated that the percentage of IFN-γ^+^ CD8^+^ T cells was significantly increased in the RFA + Abs group when compared with that in the RFA group (Figures 5A,B).

### Long-Term Outcomes

**FIGURE 6:**
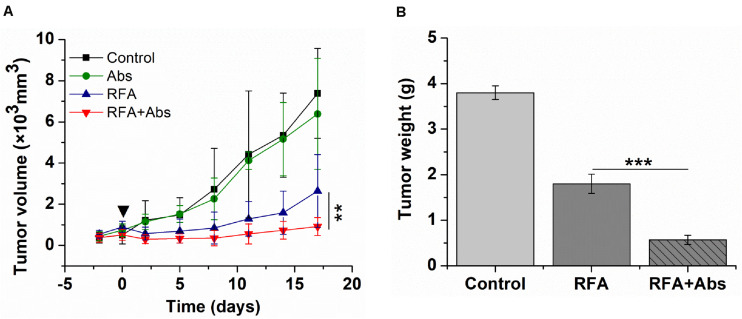
Long-term outcomes after different treatments in the CT26 tumor model. **(A)** the tumor growth rate in the RFA + Abs group was lowest when compared to RFA alone group (*P* < 0.01) and Abs alone group, and there was no significant difference between Abs alone group and the control group, **(B)** the tumor weight in RFA + Abs group was 3.0-fold lower than that in RFA group. ***P < 0.001, **P < 0.01.

As shown by the tumor growth curves, the tumor growth rate in the RFA + Abs group was lowest when compared to the RFA alone group (*P* < 0.01) and Abs alone group (*P* < 0.01). There was no significant difference between the Abs alone group and the control group (*P* > 0.05) (Figure 6A). After finishing this experiment, the weight of the tumor was measured, and the results showed that the tumor weight in the RFA + Abs group was much lower than that in the RFA group (*P* < 0.001) (Figure 6B and Supplementary Figures S4A,B).

## Discussion

The results of this study demonstrated that RFA promoted the migration of MDSCs to peripheral blood, and the proportion of MDSCs in peripheral blood at 3 days after RFA was 1.9-fold higher than that before RFA. Moreover, the ratio of CD4/CD8 was declining within 8 days after RFA, indicating the dysfunction of the effector T cells to some extent (16–19). These findings are similar to those of previous studies examining the relationship between tumor-associated antigen (TAA)-specific T cells and the frequency of MDSCs. For example, a report from Shuichi Kaneko et al. indicates that the number of TAA-specific T cells after RFA was inversely correlated with the frequency of CD14^+^HLA-DR^–/low^ MDSCs in peripheral blood (20). These previous studies were confined to investigating the peripheral immune response rather than the TME. The results from this study go well beyond previous findings, as current data showed that RFA further promoted the infiltration of MDSC in TME. The phenomenon may be due to heat-induced injury, which can lead to the release of a large amount of pro-inflammatory factors.

As the key pro-inflammatory factor, the level of IL-6 was analyzed in our study, and the results showed that the level of IL-6 was increasing within 8 days after RFA alone, when compared with the control group. This data was consistent with the result of cytoplasmic staining that the expression level of IL-6 in RFA alone was higher than that in other groups. Previous studies have shown that several factors, such as IL-6, trigger different signaling pathways in MDSCs that mainly involve the signal transducer and activator of transcription 3 (STAT3). Signal transducer and activator of transcription 3 regulates the expansion of MDSCs by stimulating myelopoiesis and inhibiting myeloid-cell differentiation, and it also promotes MDSCs survival by inducing the expression of Myc, cyclin D1, and B-cell lymphoma XL (BCL-XL), eventually inducing the expansion of MDSCs in TEM (11, 21). Similar studies also demonstrated that IL-6, a downstream mediator of the IL-1β, induced expansion of MDSCs, and IL-1 receptor (IL-1R)-deficient mice have a delayed accumulation of MDSCs and delayed primary and metastatic tumor progression (22–24).

In the present study, the roles of MDSCs in tumor progression after pRFA was further evaluated by the targeted depletion of MDSCs via the Gr-1 (RB6-8C5) antibody. Results showed that the combination of the antibody with pRFA decreased the accumulation of MDSCs in TEM after pRFA. The mechanism for Gr-1 antibody-mediated depletion could include the killing of MDSCs by complement-dependent and -independent cytotoxicity (25). Additionally, previous studies also demonstrated that the Gr-1 antibody induces signals leading to myelopoiesis, macrophage differentiation, neutrophil apoptosis, and abrogation of MDSC activity (26, 27). Meanwhile, the tumor growth was also observed in different treatment groups, and the results found that the combination of the antibody with pRFA delayed tumor progression in this study. This may be associated with reversing immunosuppression via the targeted depletion of MDSCs, which was to some extent confirmed by the ELISA of cytokines in this study. The results from the ELISA assay showed that the level of TGF-β had been increasing within 8 days in the RFA group, and the level of TGF-β at 8 days after RFA alone was 1.23-fold higher than that before RFA. However, in the RFA + Abs group, the level of TGF-β had been declining within 8 days, and the level of TGF-β at 8 days after RFA + Abs was 1.63-fold lower than that before RFA. The cytoplasmic staining of TGF-β also displayed that the expression level of TGF-β in the RFA + Abs group was much weaker than that in the RFA alone group. These findings were consistent with the results from Berzofsky et al. that TGF-β derived mainly from CD11b^+^Gr-1^+^ cells (MDSCs) in tumor-bearing mice induced the down-regulation of tumor immunosurveillance and tumor recurrence (28).

Additionally, as the potent antigen-presenting cells, DCs in the TEM was also equally important (29), so tumor-infiltrating DCs before and after RFA or RFA + Abs was analyzed in this study. The results showed that RFA alone increased tumor-infiltrating DCs when compared with the control group, which was consistent with previous findings, however, the tumor progression in the RFA group could not be inhibited. Previous studies have demonstrated that total RFA treatment resulted in enhanced systemic antitumor T-cell immune responses and tumor regression that was associated with increased DC infiltration (7). The difference may be related to the heterogeneity in immune responses, which firstly should be considered due to the difference of tumor mutation burden (TMB) in different tumor models in both experiments (30–32). Additionally, take an example in this study, the boundary of recurrent tumor after pRFA alone was more irregular in 4T1 tumor models than that in CT26 tumor models, which displayed worse biology behavior after pRFA in 4T1 tumor models (data not shown); and the hepatic metastasis was showed after pRFA alone in 4T1 tumor models (Supplementary Figure S5). Moreover, the pre-RFA tumor burden may have certain effects on the prognosis of treatment in these two experiments, and the pre-RFA tumor size in this study (the average diameter was 12 mm) was larger than that in the previous report (the average diameter was 7.8 mm). Accumulating evidence showed that the relationship between the intensity of the immune response and pretreatment tumor burden was vital for achieving effective immunotherapy (33). John Wherry et al. have demonstrated that immunotherapy failure or success in many patients was not solely due to the ability to induce immune reinvigoration, rather it resulted from the balance between T-cell reinvigoration and tumor burden. They also confirmed a calibration of immune responses to antigen burden and raise the possibility that even robust CD8 T-cell reinvigoration by immunotherapy may be clinically ineffective if the tumor burden is high.

In addition, T cell exhaustion as a state of T cell dysfunction that arises during tumor progression, should not be ignored. Past studies have demonstrated that naive T cells are primed by antigen, costimulation, and inflammation, and then differentiate into effector T cells during the initial inflammatory response. However, T cells are accompanied by a progressive increase in the amount and diversity of inhibitory receptors expressed, such as PD-1/PD-L1, LAG-3. T cells could progress through stages of dysfunction or be completely eliminated, leading to the loss of T-cell responses when inflammatory response or antigens persist (34). Concerning the relationship between T-cell responses and RFA treatment, recent reports have showed that RFA induced a marked decrease of the functional TILs in CT26 tumor tissues at 8 days after RFA, which may be associated with the persistent intracellular necrotic debris (as an effective *in situ* antigen) from thermal ablation (35). In the current study, similar results also showed that the amount of IFN-γ^+^ CD8^+^ TILs was at a low level at 8 days after RFA alone, which may be attributed to the persistence of *in situ* antigens and the elevated extracellular potassium (34, 36). Yet, the amount of IFN-γ^+^ CD8^+^ TIL increased significantly after RFA, when combined with the GR-1 antibody, which may be due to the activation of T cell and the inhibition of T cell exhaustion because of the depletion of MDSCs. The results from long-term follow-up also showed that RFA treatment further delayed the tumor progression at different degrees when combined with the depletion of MDSCs in CT26 and 4T1 tumor models. Taken together, tumor-infiltrating MDSCs could be associated with tumor recurrence after RFA and recruiting MDSCs after pRFA could have an influence on the function of DC and induce T cell exhaustion.

While these observations are compelling, it must be acknowledged that there were several limitations to this study. Firstly, although murine CT26 and 4T1 tumor models are the classic tumor model for immunologic studies, only two tumor cell lines were used in this study. Future studies will explore whether similar observations after RFA would occur in other tumor models. Secondly, GR-1 antibodies play an important role in targeted depletion of MDSCs in mice. However, the phenotype of MDSCs is complex in humans. In other words, the depletion of MDSCs by GR-1 antibodies could not be achieved in humans. Therefore, further studies will focus on exploring how to clear MDSCs in humans from different perspectives as a way of achieving better therapeutic efficacy after RFA.

In conclusion, the current study found that pRFA accelerates residual tumor progression through increasing tumor-infiltrating MDSCs and reducing T-cell-mediated anti-tumor immune responses. This study also indicated that the standardization of RFA techniques is essential to achieving complete ablation of a tumor and in preventing tumor progression. When taken together, the findings of this study could provide a potential approach for delaying tumor recurrence caused by pRFA and shows the importance of the standardization of RFA techniques.

## Data Availability Statement

All datasets presented in this study are included in the article/Supplementary Material.

## Ethics Statement

The animal study was reviewed and approved by the Institutional Animal Care and Use Committee of Peking University Cancer Hospital.

## Author Contributions

HW, WY, and HL designed the study, analyzed the data, and wrote the manuscript. HW, SL, A-NJ, MZ, YH, and SW conducted the experiments. HL and WY read and approved the final manuscript. HW and SL were equally responsible for all parts of the manuscript. All authors contributed to the article and approved the submitted version.

## Conflict of Interest

The authors declare that the research was conducted in the absence of any commercial or financial relationships that could be construed as a potential conflict of interest.
