# Supplementary material for: Palliative Radiofrequency Ablation Accelerates the Residual Tumor Progression Through Increasing Tumor-Infiltrating MDSCs and Reducing T-Cell-Mediated Anti-Tumor Immune Responses in Animal Model
Source: Front Oncol. 2020 Sep 4;10:1308. doi: 10.3389/fonc.2020.01308 (PMC7498645; doi:10.3389/fonc.2020.01308)
Supplement: Supplementary file 1 [file Data_Sheet_1.pdf]

Supplementary materials

Animals and tumor model

The experimental animal protocol was first approved by the Institutional Animal Care and Use Committee of Peking University Cancer Hospital. Babl/c mice (6-8weeks, Beijing Weitonglihua Medical Experimental Animal Center, Beijing, China) were used and housed in a specific pathogen-free facility in this study. For all experiments and procedures, animals were anesthetized by using isoflurane inhalation.

CT26 and 4T1 tumor cells were cultured in RPMI 1640 medium (Hyclone, USA) with 10% FBS, 100 g·mL-1 streptomycin and 100 U·mL^{-1} penicillin at 37°C in an atmosphere containing 5% CO_2. To develop the tumor model, 2×10^6 CT26 cells were subcutaneously injected into the flanks of the mice. Animals were monitored every 2–3 days to measure tumor growth. Tumor diameters were measured every day in the longitudinal and transverse directions with mechanical calipers (W.H., J.A.N. and W.S with 5, 2 and 5 years of experience in animal experiments). Tumor volume was calculated as $D \times d^2/2$, where D is the longest diameter of tumor and d is the shortest diameter of tumor. When tumors reaches an approximate volume of 500 cm^3, the mice were randomly assigned to one of the treatment or groups. Mice were euthanized when tumor largest diameters reached 2cm, in accordance with animal protocols.

Radiofrequency ablation procedure

The standard monopolar RFA was applied using a 480-kHz RFA generator (Model CC-1-220; Valleylab, Tyco Healthcare, USA). To complete the RFA circuit, the animal was placed on a conventional metallic grounding pad (Cosman Medical, Inc. USA). Contact was ensured by shaving and cleaning the animal’s back and by liberally applying electrolytic contact gel. Initially, the 0.7-cm tip of a 17-gaugeelectrically insulated electrode (ACT1507 electrode; Valleylab, Tyco Healthcare) was placed just beneath the skin for partial tumor ablation. The mean impedance and mean temperature before RF ablation were approximately 300 ohms and 27°C, respectively. To investigate the influence of RFA on CD4+ T cells, CD8+ T cells and MDSCs in peripheral blood, sufficient ablation were performed for less than 70 seconds at approximately 90°C (as

measured with the RF needle) by using a mean power output of 5 W. For tumor growth studies, tumors were treated with a similar power output but to a maximum temperature of 70°C for approximately 20 seconds in the center of the tumor. The shorter treatment regimen resulted in a smaller treatment volume and a greater reliance on systemic immune responses for tumor regression.

The effect of MDSCs on tumor immunity after the combination of RFA with antibody

To investigate the level of TGF-β and IL-1β in the tumor tissue after different treatments, immunohistochemical staining was performed. Firstly, tumor tissues were fixed in 10% formalin and embedded in paraffin in accordance with standard procedures. Sections were stained with anti-TGF-β/ IL-1β antibodies. Observations were performed with a microscope for immunohistochemical staining analysis, and representative digital images were obtained.

Long-term outcomes

Twenty mice bearing 4T1 tumor were used in this phase. Tumors measuring 10-12 mm size were randomized into 4 experimental groups for tumor growth (n=5/group for 4T1). These groups included: (a) control (PBS only), (b) Abs, (c) RFA, (d) RFA+Abs. The mice receiving PBS without RFA or Abs was used as the control. After finishing this experiment, tumors were harvested from mice, and then the weight of tumor was measured.

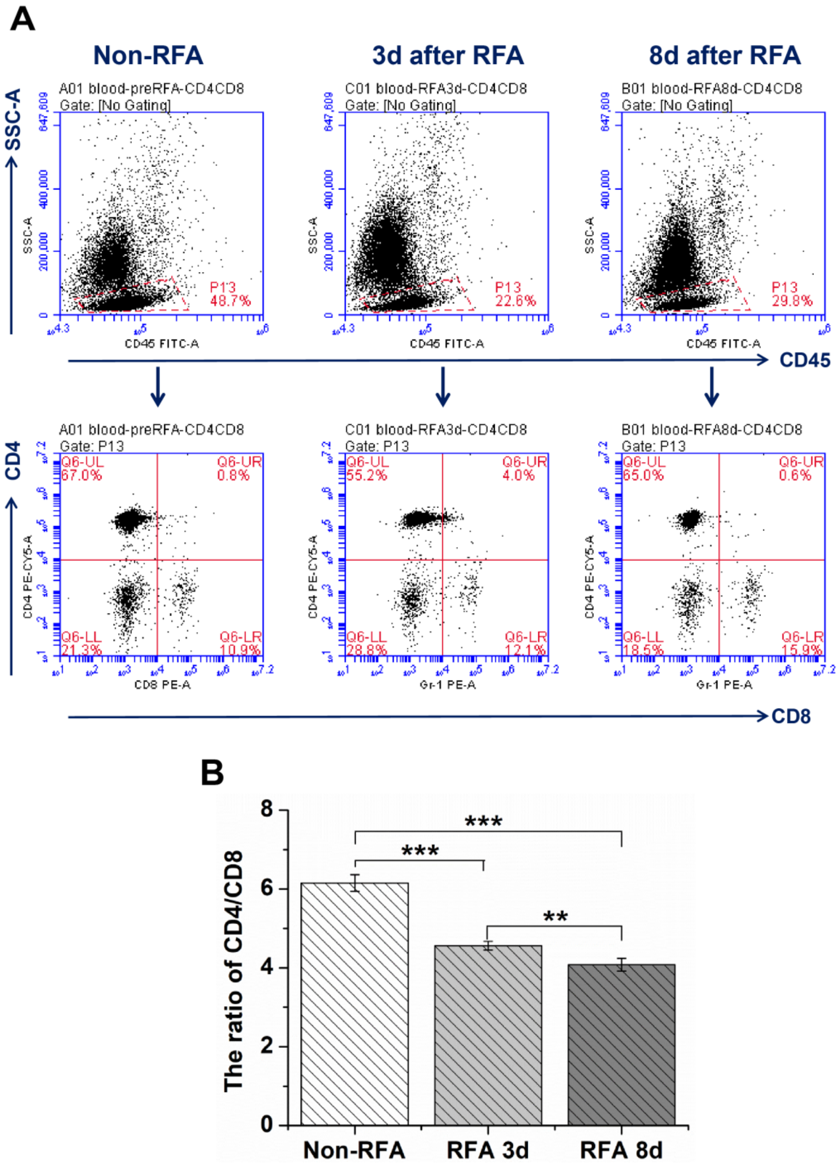

Figure S1

Figure S1: The flow cytometry analysis for $CD4^+$ and $CD8^+$ T cells in CT26 tumor models. (A) The flow cytometry analysis for $CD4^+$ and $CD8^+$ T cells was performed on blood cells from mice bearing tumour treated with different treatment groups (Non-RFA, 3d after RFA, 8d after RFA); (B) The ratio of CD4/CD8 from the quantitative analysis of flow cytometry, ***P＜0.001, **P＜0.01.

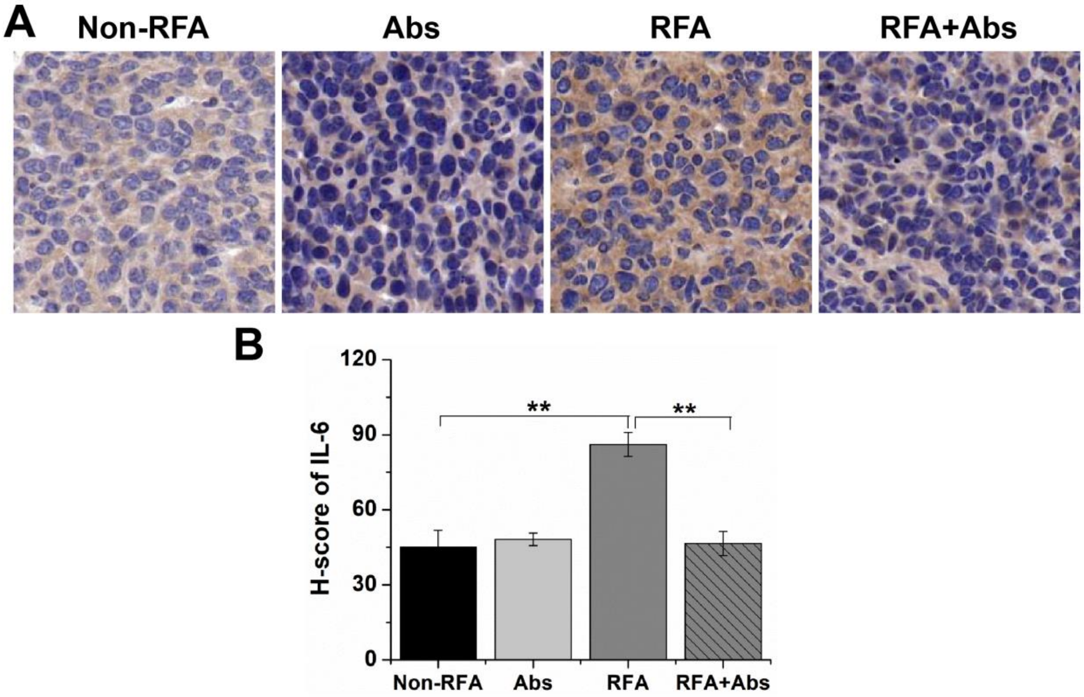

Figure S2

Figure S2: The immunohistochemical staining for intratumoral level of IL-6 in CT26 tumor models. (A) the representative images of immunohistochemical staining for intratumoral level of IL-6 from different treatment groups (Non-RFA, Abs, RFA and RFA+Abs); (B) the quantitative analysis of intratumoral level of IL-6 from different treatment groups, ***P＜0.001, **P＜0.01.

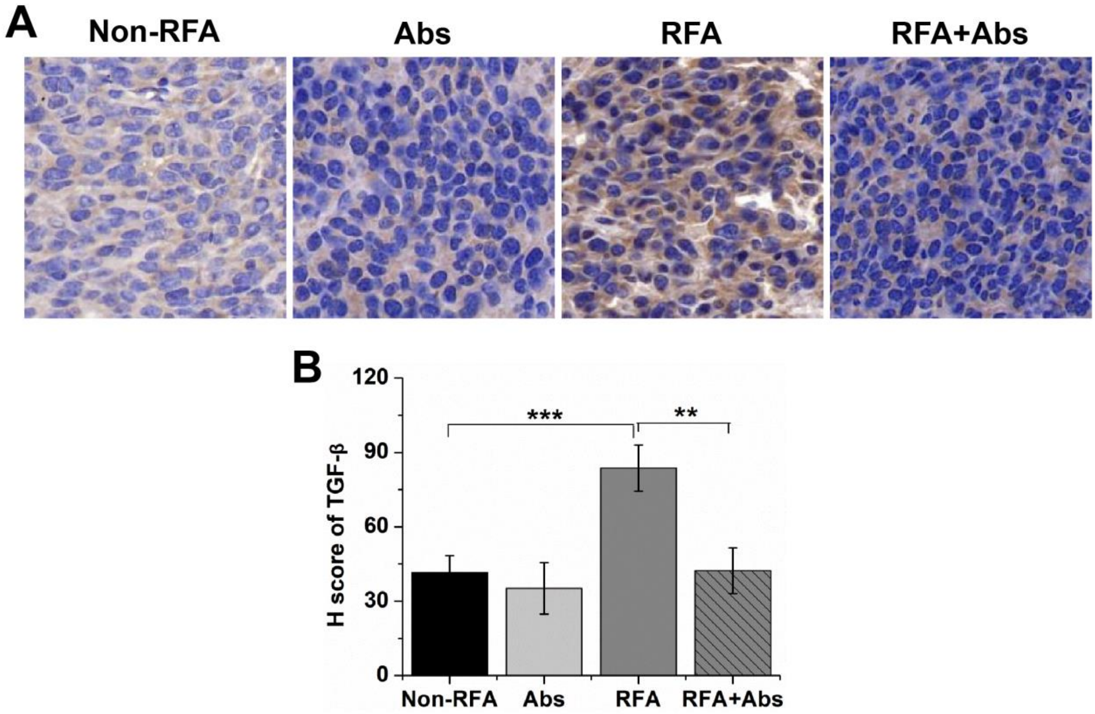

Figure S3

Figure S3: The immunohistochemical staining for intratumoral level of TGF-β in CT26 tumor models. (A) the representative images of immunohistochemical staining for intratumoral level of TGF-β from different treatment groups (Non-RFA, Abs, RFA and RFA+Abs); (B) the quantitative analysis of intratumoral level of TGF-β from different treatment groups, ***P＜0.001, **P＜0.01.

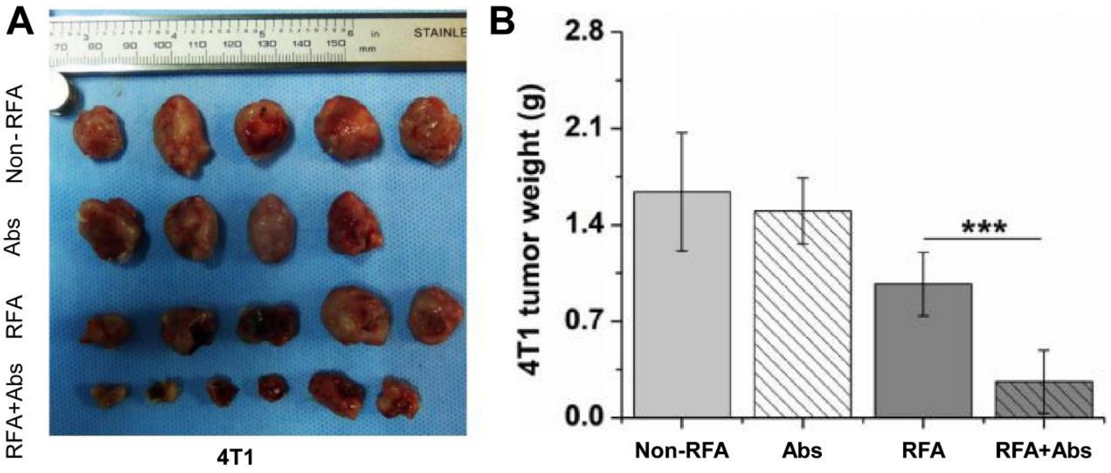

Figure S4. Long-term outcomes after different treatments in 4T1 tumor models. (A) The representative picture after different treatment groups. (B) the final tumor weight at different treatment groups (Control, Abs, RFA and RFA+Abs), ***P＜0.001.

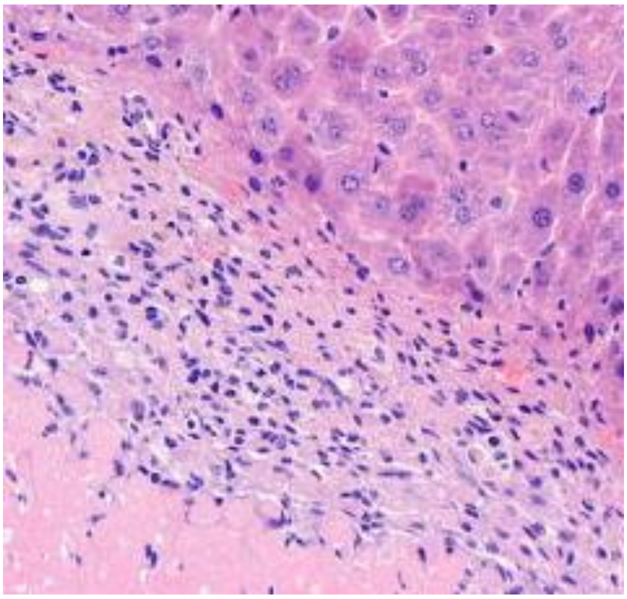

Figure S5. the hepatic metastasis was showed after pRFA alone.
